# LncRNA SNHG16 contributes to tumor progression via the miR-302b-3p/SLC2A4 axis in pancreatic adenocarcinoma

**DOI:** 10.1186/s12935-020-01715-9

**Published:** 2021-01-12

**Authors:** Hao Xu, Xin Miao, Xin Li, Haofei Chen, Bo Zhang, Wence Zhou

**Affiliations:** 1 The Second Department of General Surgery, The First Hospital of Lanzhou University, Lanzhou, Gansu China; 2 The First Clinical Medical School of Lanzhou University, Lanzhou, Gansu China; 3 Institute of Hepatopancreatobiliary Surgery of Gansu, Lanzhou, Gansu China; 4 State Key Laboratory of Veterinary Etiological Biology & OIE/National Foot and Mouth Disease Reference Laboratory & Key Laboratory of Animal Virology of the Ministry of Agriculture, Lanzhou Veterinary Research Institute, Chinese Academy of Agricultural Sciences, Lanzhou, Gansu China

**Keywords:** Pancreatic adenocarcinoma, SNHG16, miR-302b-3p, SLC2A4

## Abstract

**Background:**

It has been reported that the lncRNA SNHG16 has significantly increased expression in pancreatic adenocarcinoma (PC). However, the functions and mechanisms of SNHG16 are not clear. The aim of this study was to explore the effects of SNHG16 on PC.

**Methods:**

qRT-PCR analysis was applied to detect the expression levels of SNHG16, miR-302b-3p and SLC2A4 in PC tissues and cells. CCK8 and EdU assays were used to evaluate the proliferation of PC cells. Transwell assays were used to assess PC cell migration and invasion. Apoptosis was evaluated by flow cytometry, and the expression of apoptosis-related proteins (including Bax, Bcl-2, cleaved caspase-3 and cleaved caspase-9) was tested by western blotting. The interactions between miR-302b-3p and SNHG16 or miR-302b-3p and the 3’UTR of SLC2A4 mRNA were clarified by a dual luciferase reporter assay and RNA immunoprecipitation.

**Results:**

SNHG16 expression was significantly elevated in PC tissues and cell lines and was associated with poor prognosis of PC patients. Knockdown of SNHG16 reduced PC cell proliferation, migration and invasion. SNHG16 acted as a sponge to regulate miR-302b-3p expression in PC cells. In addition, miR-302b-3p targeted SLC2A4 directly.

**Conclusions:**

SNHG16 promoted the progression of PC via the miR-302b-3p/SLC2A4 axis and was expected to be a potential target for the early diagnosis and treatment of PC.

## Introduction

Pancreatic adenocarcinoma (PC) is one of the most severe gastrointestinal malignancies and is the fourth most common cause of cancer-related death [1]. Due to the lack of specific and accurate diagnostic methods, PCs are mostly diagnosed in late stages. Patients with advanced PC cannot undergo surgical resection and can only receive chemotherapy, but the effect is not significant [2]. Because PC results from a biological process involving multiple steps, there is no clinically sensitive indicator for early diagnosis or effective treatment approach [3]. Therefore, research on diagnostic markers and targeted therapies to gradually inhibit the progression of PC has become the focus of attention in the treatment of PC.

It has been proven that only 2% of the genome sequence is capable of coding proteins, whereas more than 95% of transcripts are identified as noncoding RNAs [4]. Long noncoding RNAs (lncRNAs) are a class of noncoding RNAs that have no protein-coding ability and are longer than 200 nt. Emerging evidence demonstrates that lncRNAs are involved in numerous malignancies, including PC [5–7]. For example, the lncRNA HOTTIP contributes to PC by enhancing Wnt/β-catenin pathway activity via binding to WDR5 [8]. However, it was reported that LINC01197 was downregulated in PC tissues, which inactivated the Wnt/β-catenin pathway by interfering with β-catenin binding to TCF4 in PC cells [9].

Recently, small nucleolar RNA host gene 16 (SNGH16) was gradually discovered to be involved in tumorigenesis in many cancer types [10, 11]. SNHG16 is also widely regarded as an essential oncogene [12]. In hepatocellular carcinoma, SNHG16 is highly expressed in HCC-resistant tissues and promotes HCC cell viability and autophagy while suppressing apoptosis by regulating the miR-23b-3p/EGR1 pathway [13]. However, the exact roles of SNHG16 in PC have not yet been clarified.

In this study, we aimed to explore the roles of the lncRNA SNHG16 with functional and mechanistic impact PC. By detecting SNHG16 expression in paired PC tissues and cell lines, we found that the lncRNA SNHG16 was significantly overexpressed in PC. In addition, elevated SNHG16 played an oncogenic role in PC cell proliferation, migration, invasion and apoptosis. Mechanistic investigations revealed that SNHG16 acted as a sponge of miR-302b-3p, which reversed SNHG16-induced proliferation by targeting SLC2A4 in PC.

## Materials and methods

### Tissue collection

PC samples and corresponding adjacent noncancerous tissues were collected from PC patients who underwent surgery at the First Hospital of Lanzhou University. The study was approved by the ethics committee of the First Hospital of Lanzhou University, and all included patients signed informed consent forms. Clinical tissue specimens were collected and the clinicopathologic characteristics were presented in Supplementary materials.

### Cell culture

The human normal pancreatic duct epithelial cell line (HPY-Y5) and pancreatic adenocarcinoma cell lines (including BxPC3, Panc-1, MIA Paca-2 and SW1990) were purchased from the Chinese Academy of Sciences, Shanghai, China. All PC cells were cultured in high-glucose Dulbecco’s modified Eagle’s medium (DMEM) containing 10% fetal bovine serum (Gibco, USA) in a sterile incubator with 5% CO_2_ at 37 °C.

### RNA isolation, reverse transcription and qRT-PCR assay

Total RNA was extracted from tumor tissues and cells with TRIzol reagent (Invitrogen, USA) according to the manufacturer’s instructions. cDNA was generated with a reverse transcription kit from Takara (Dalian, China). Amplification reactions included 2 µl of Mix, 2 µl of RNA and 6 µl of ddH_2_O. The thermal cycling protocol was as follows: 37 °C for 15 min, 85 °C for 5 s and 4 °C for 15 min. The expression of SNHG16 and SLC2A4 was detected by using a SYBR Green Real-Time PCR Kit (Qiagen, Germany). miR-302b-3p expression was analyzed with a Hairpin-it^™^ miRNA qPCR kit (GenePharma, China). Amplification of the transcripts involved an initial denaturation step at 95 °C for 2 min followed by 40 cycles at 95 °C for 5 s, 55 °C for 30 s, and 72 °C for 30 s. The mRNA and miRNA expression data are presented as fold changes in the mRNA/miRNA abundance normalized to β-actin or U6 snRNA expression. The primer sequences used in this study are presented in Table 1.

**Table 1:** Primer sequences used for qRT-PCR

Gene	Primer sequences
SNHG16	
F	5ʹ-CAGAATGCCATGGTTTCCCC-3ʹ′
R	5ʹ-TGGCAAGAGACTTCCTGAGG-3ʹ
miR-302b-3p	
Loop	5′-GTCGTATCCAGTCCAGGGACCGAGGACTGGATACGACCTACTA-3′
F	5′-GCGTAAGTGCTTCCATGTT-3′
R	5′-TCCAGGGACCGAGGA-3′
SLC2A4	
F	5′-TGGCTGGGTTCTCCAACTG-3′
R	5′-CTGGAAACCCATGCCAATG-3′
β-actin	
F	5′-ACCGAGCGCGGCTACAG-3′
R	5′-CTTAATGTCACGCACGATTTCC-3′
U6 snRNA	
F	5′ -CTCGCTTCGGCAGCACA-3′
R	5′-AACGCTTCACGAATTTGCGT-3′

### Plasmids construction and cell transfection

The miR-302b-3p mimics, miR-302b-3p inhibitors, small hairpin RNA of SLC2A4 (sh-SLC2A4) and corresponding negative controls (NC mimic or sh-NC) were purchased from RiboBio, Guangzhou, China. The inhibitors and shRNAs were transfected into BxPC3 and Panc-1 cells using Lipofectamine 3000 (Invitrogen, USA) according to the manufacturer’s directions. The transfection efficiencies were detected by qRT-PCR at 48 h post transfection. Transfected cells were combined for the subsequent experiments.

### Construction of stably transfected cells

The lentiviral particles LV-shSNHG16 and LV-sh-NC were purchased from GenePharma, China. The lentiviral particles were used to directly infect BxPC3 and Panc-1 cells in the presence of 8 µg/ml polybrene (Sigma-Aldrich) to construct the stably transfected cells. Puromycin (Invitrogen, USA) was added to the medium 48 h after infection and maintained for 2 weeks to select stably transfected cells (BxPC3/Panc-1-sh-SNHG16 or BxPC3/Panc-1-sh-NC).

### EdU assay

The reagents used in the EdU assay were purchased from RiboBio (Guangzhou, China). PC cells were seeded in confocal dishes at a density of 3 × 10^5^. Then, 4% paraformaldehyde (Beyotime, China) was used to fix the cells for 10 min. Then, 1% Triton (Beyotime, China) was used to clear the cells for 5 min after three washes with PBS. Subsequently, the cells were incubated with dyeing agent for 30 min in the dark, stained with DAPI (Olympus, Tokyo, Japan) and incubated for 5 min at 37 °C. Images were acquired using a microscope at a magnification of 400×.

### Wound healing assay

PC cells were seeded in 6-well plates and grown to 70–80% confluence. A wound was created by scraping with a 100 µl pipette tip. The remaining cells were cultured in serum-free DMEM for 48 h. Images of migration were acquired at 0 and 48 h after scraping.

### Migration and invasion assays

Cells were suspended in serum-free DMEM. One hundred microliters of the cell suspension was seeded in the upper compartment of 8 µm transwell chambers in a 24-well culture plate (Corning, USA) for 48 h, and 500 µl of DMEM supplemented with 10% FBS was added to the lower compartment of the chambers. Chambers with inserts uniformly coated with Matrigel (BD Biosciences) were used for the invasion assay. The transwell chambers were fixed with 4% paraformaldehyde for 15 min and stained with 0.1% crystal violet for 30 min at room temperature. Then, cells on the upper surface of the inserts were wiped off with a cotton swab. Stained cells were washed with PBS and observed with a microscope. The capacities for cell migration and invasion were assessed by determining the average numbers of stained cells in 5 regions.

### Luciferase reporter assay

Full-length SNHG16 (WT or Mut) was synthesized and cloned into the pGL3-–Promoter vector (Promega, USA). Dual luciferase reporter assays were conducted in BxPC3 and PANC-1 cells cotransfected with pGL3-Promoter-SNHG16 (WT or Mut) and NC mimics or miR-302b-3p mimics. Luciferase activity was analyzed with a dual luciferase reporter assay system (Promega) according to the manufacturer’s instructions.

### Western Blot analysis

Total protein was extracted from cells with RIPA buffer (Beyotime, China). The concentration of total protein was determined with a BCA protein assay kit (Beyotime, China). Thirty micrograms of total protein was separated on 6%, 8% or 10% SDS-PAGE gels and was then transferred to PVDF membranes (Millipore, USA). After blocking with 5% skim milk for 2 h, membranes were incubated overnight with a specific primary antibody at 4 ℃. The specific primary antibodies (including antibodies specific for Bax, Bcl-2, cleaved caspase-3, and cleaved caspase-9) were purchased from Proteintech Group, and the concentration of these antibodies applied in this study was 1:500. Specific primary antibodies (including antibodies specific for ICAM-1, VCAM-1 and MMP9) were purchased from Abcam, and the concentration of these antibodies applied in this study was 1:1000. Next, membranes were incubated with the corresponding secondary antibodies for 2 h at room temperature. The protein signals were visualized with ECL western blotting substrate (Tanon, Shanghai, China).

### Statistical analysis

All statistical analyses were performed with SPSS 22.0 software (Chicago, USA) and Prism version 7.0 software (California, USA). Data are presented as the mean ± SEM values. An unpaired *t*-test was performed to compare the means of two groups. One-way ANOVA and the Bonferroni test for multiple comparisons were applied to analyze differences among two or more groups. Spearman correlation analysis was performed to detect correlations between SNHG16, miR-302b-3p and SLC2A4 levels. Significant differences were defined as those with *P *< 0.05.

## Results

### LncRNA SNHG16 is elevated in PC tissues and cell lines

**Fig. 1:**
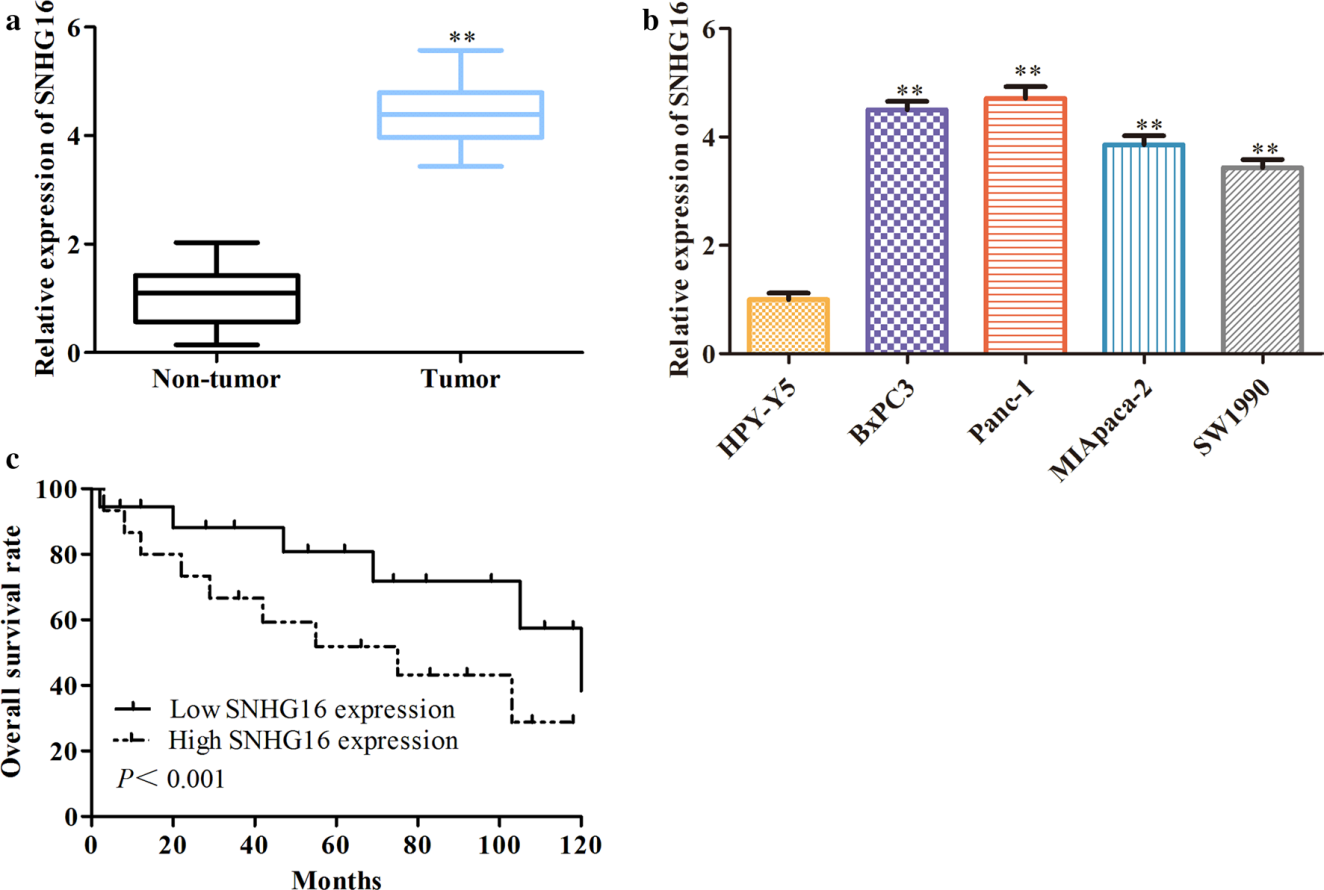
LncRNA SNHG16 expression is elevated in PC tissues and cell lines. **a** The expression levels of SNHG16 in PC tissues and adjacent normal tissues were detected by qRT-PCR. **b** The expression of SNHG16 in the normal pancreatic duct epithelial cell line HPY-Y5 and pancreatic adenocarcinoma cell lines (BxPC3, Panc-1, MIA Paca-2 and SW1990). **c** Kaplan-Meier overall survival analysis of the relationship between SNHG16 expression and the survival time of PC patients. **P < 0.01

In this study, the expression level of SNHG16 in human PC tissues was first evaluated by qRT-PCR. Compared with that in pair-matched adjacent normal samples, SNHG16 expression was significantly increased in PC tissues (Fig. 1a). Moreover, the expression of SNHG16 was upregulated in four PC cell lines (BxPC3, Panc-1, MIA Paca-2 and SW1990) compared with HPY-Y5 cells (Fig. 1b). In addition, Kaplan-Meier analysis indicated that PC patients with high SNHG16 expression showed shorter overall survival times than those with low SNHG16 expression (Fig. 1c). The above results indicate that elevated SNHG16 expression might play a critical role in the progression of PC.

## SNHG16 affects PC cell proliferation and apoptosis

**Fig. 2:**
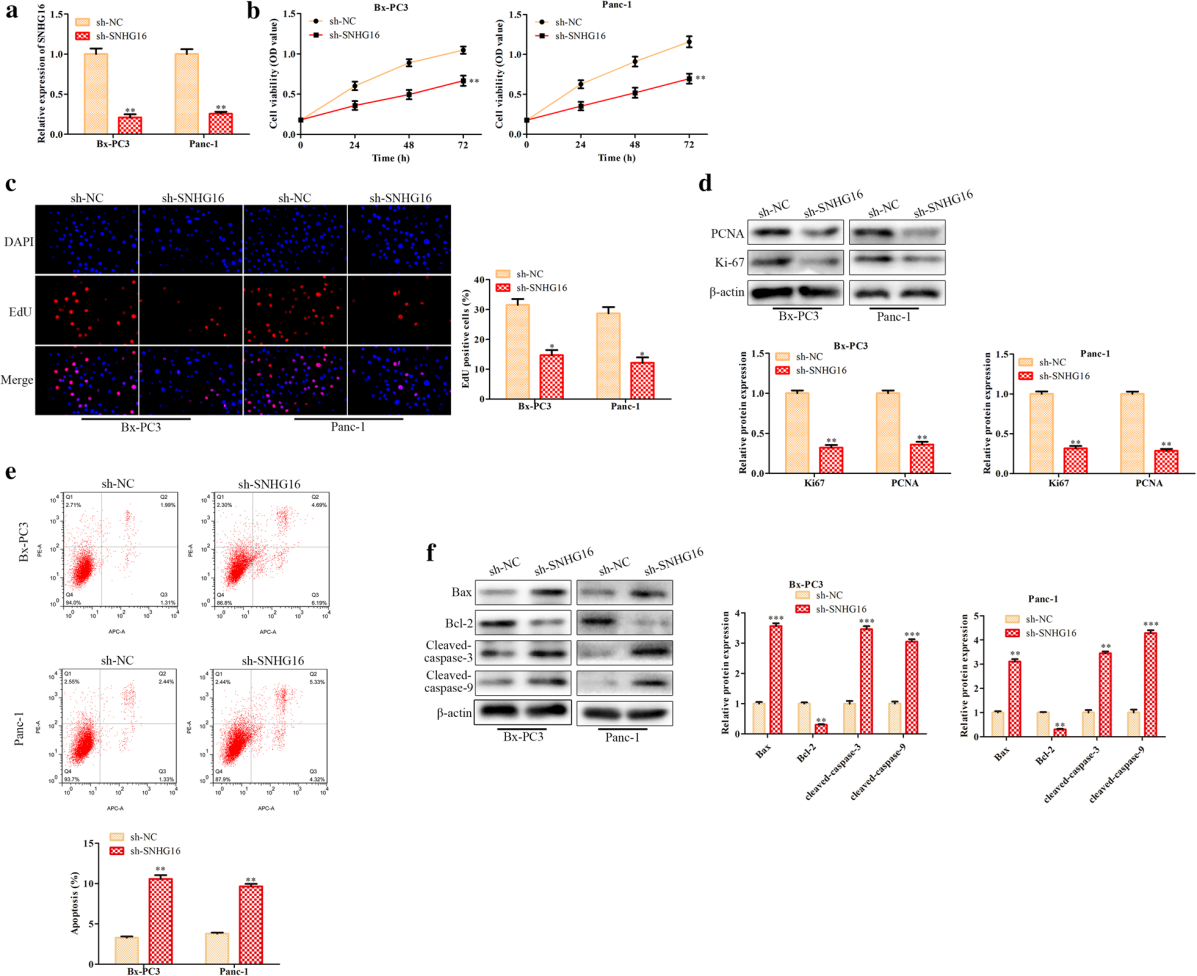
Knockdown of SNHG16 inhibits the proliferation of PC cells and induces their apoptosis. BxPC3 and Panc-1 PC cells were transfected separately with sh-SNHG16 or sh-NC. **a** Relative expression levels of SNHG16 in PC cells were detected by qRT-PCR. **b** The viability of PC cells was analyzed by a CCK8 assay. **c** The proliferation ability of PC cells was assessed by an EdU assay. **d** The cell proliferation markers PCNA and Ki-67 were detected by western blotting. **e** The apoptosis rate of PC cells was analyzed by flow cytometry. **f** Western blot analysis of the apoptosis-related proteins Bax, Bcl-2, cleaved caspase-3 and cleaved caspase-9. β-Actin was used as the internal control. *P < 0.05, **P < 0.01, ***P < 0.001

To explore the roles of SNHG16 in the PC process, we established BxPC3 and Panc-1 cells with stable silencing of SNHG16, which were named sh-SNHG16 cells (Fig. 2a). Next, CCK8 and EdU assays were employed to evaluate the proliferation of PC cells, and knockdown of SNHG16 was found to inhibit BxPC3 and Panc-1 cell proliferation (Fig. 2b, c). Consistent with this finding, the protein levels of the cell proliferation markers PCNA and Ki-67 were decreased with SNHG16 silencing (Fig. 2d). Then, flow cytometric analysis of cell apoptosis demonstrated that SNHG16 knockdown increased the proportion of apoptotic PC cells (Fig. 2e), and western blot analysis revealed that SNHG16 knockdown promoted the expression of the apoptosis-related proteins Bax, cleaved caspase-3 and cleaved caspase-9 while inhibiting the expression of Bcl-2 (Fig. 2f). In summary, SNHG16 knockdown suppressed the proliferation of PC cells and promoted their apoptosis.

### Knockdown of SNHG16 suppresses PC cell migration and invasion

**Fig. 3:**
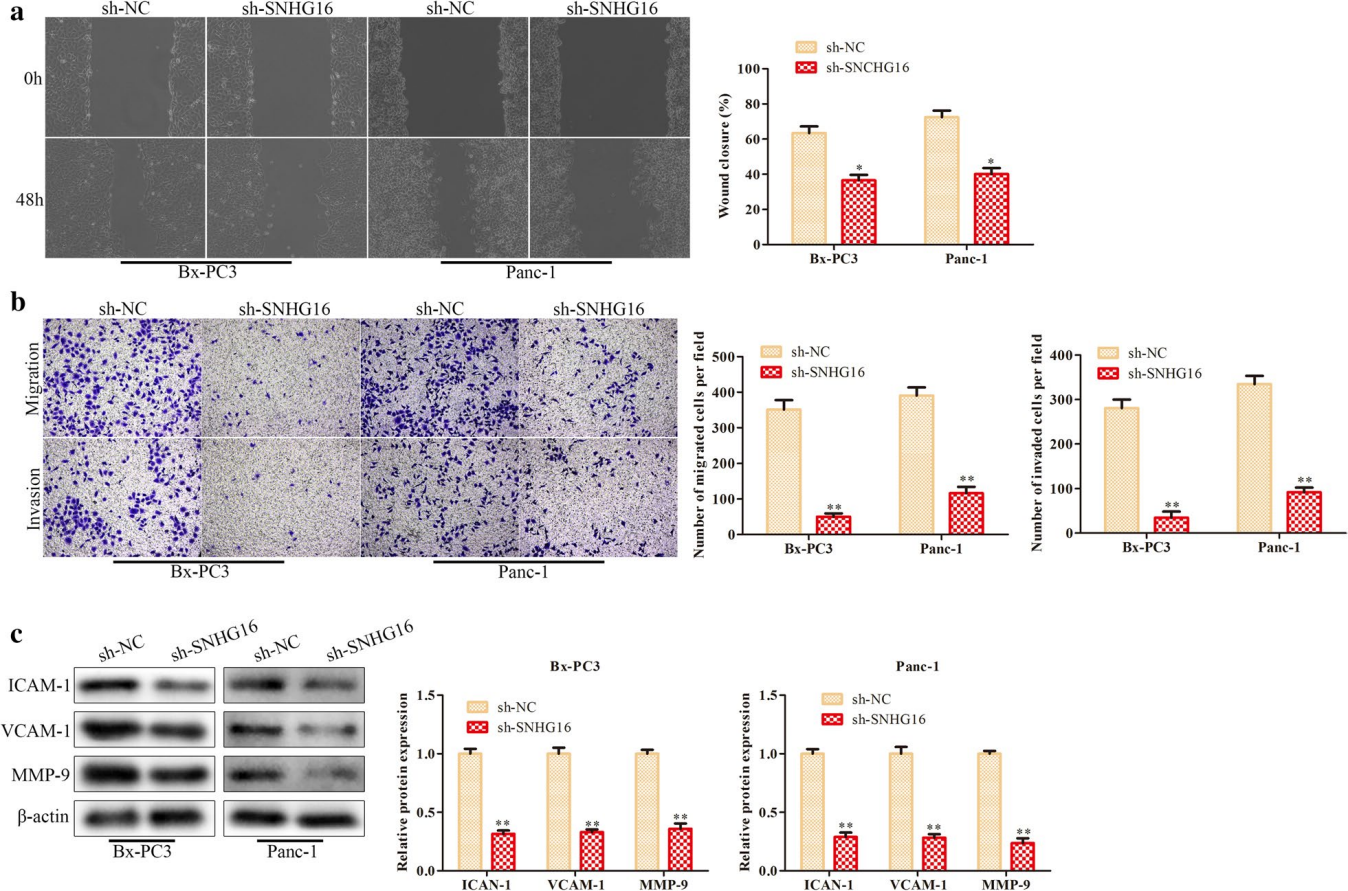
Knockdown of SNHG16 suppresses the migration and invasion of PC cells. **a** The migration ability of PC cells transfected with sh-SNHG16 or sh-NC was assessed by a wound healing assay. **b** The migration and invasion abilities of PC cells transfected with sh-SNHG16 or sh-NC were evaluated by transwell assays. **c** Western blot analysis of ICAM-1, VCAM-1 and MMP-9. β-Actin was used as the internal control. *P < 0.05, **P < 0.01

Subsequently, we examined the roles of SNHG16 in migration and invasion. The wound healing assay indicated that the motility of PC cells with stable SNHG16 silencing was significantly decreased (Fig. 3a). Transwell assays were implemented to evaluate the migration and invasion abilities of PC cells, and reduced migration and invasion abilities of PC cells with stable SNHG16 silencing were observed (Fig. 3b). The western blot results demonstrated that reduced SNHG16 expression reduced the expression of ICAM-1, VCAM-1 and MMP-9 (Fig. 3c). In general, SNHG16 knockdown suppressed PC cell migration and invasion.

### SNHG16 acts as a sponge to regulate miR-302b-3p expression in PC cells

**Fig. 4:**
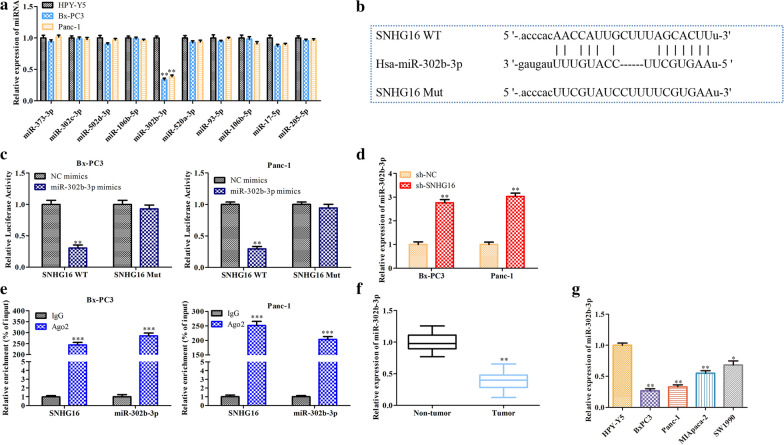
SNHG16 acts as a sponge to decrease miR-302b-3p expression in PC cells. **a** qRT-PCR analysis of SNHG16 target miRNAs in HPY-Y5, BxPC3 and Panc-1 cells. **b** Schematic diagram of the sites for binding between miR-302b-3p and SNHG16 (WT or Mut). **c** Luciferase activity of SNHG16 WT or Mut in BxPC3 and Panc-1 cells cotransfected with miR-302b-3p mimics or NC mimics. **d** Relative expression levels of miR-302b-3p in BxPC3 and Panc-1 cells with SNHG16 silencing. **e** RIP analysis of SNHG16 and miR-302b-3p enrichment in the anti-Ago2 immunoprecipitate in BxPC3 and Panc-1 cells. IgG was used as the control. **f**, **g** qRT-PCR assay of miR-302b-3p expression in PC tissues and cell lines. *P < 0.05, **P < 0.01, ***P < 0.001

It is well known that mechanistically, lncRNAs commonly act as molecular sponges of miRNAs. Previous studies confirmed that SNHG16 regulates miRNA expression [14]. Therefore, we hypothesized that SNHG16 might exert effects by interacting with miRNAs in PC. The online software ENCORI was used to predict the miRNAs that might be regulated by SNHG16, and qRT-PCR was performed to determine the expression levels of these predicted miRNAs in PC cells. Only miR-302b-3p was downregulated in PC cells (Fig. 4a), and the binding site of miR-302b-3p on SNHG16 is displayed in Fig. 4b. The expression of miR-302b-3p was increased in SNHG16-knockdown PC cells (Fig. 4c). Dual luciferase reporter assays and RIP were performed to verify whether miR-302b-3p binds to SNHG16 directly. The luciferase activity in PC cells cotransfected with SNHG16-WT and miR-302b-3p mimics was weaker than that in cells cotransfected with SNHG16-WT and NC mimics or with SNHG16-Mut and miR-302b-3p (Fig. 4d). The RIP results revealed that SNHG16 and miR-302b-3p were highly enriched in the anti-Ago2 bead immunoprecipitate compared with the IgG bead immunoprecipitate (Fig. 4e). In addition, the level of miR-302b-3p was reduced in both PC tissues and cell lines (Fig. 4f, g). Taken together, the above results prove that SNHG16 acts as a molecular sponge to regulate miR-302b-3p expression in PC cells.

### Overexpression of miR-302b-3p inhibits the proliferation, migration and invasion of PC cells and promotes their apoptosis

**Fig. 5:**
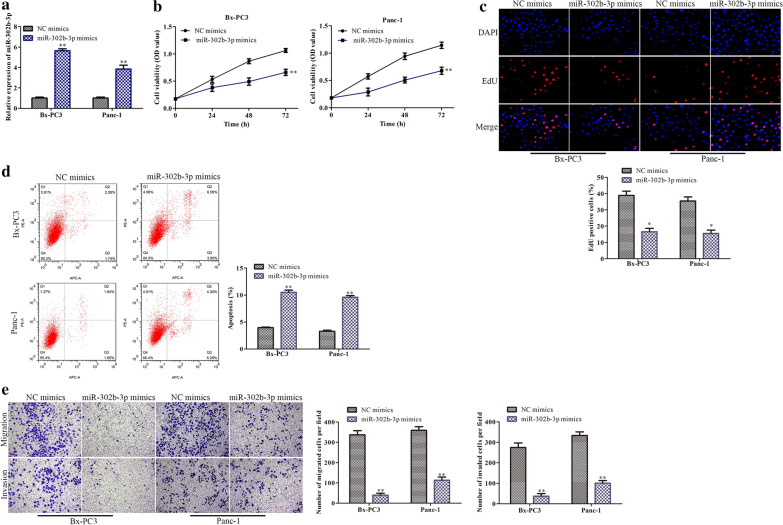
MiR-302b-3p inhibits the metastasis and induces the apoptosis of PC cells. BxPC3 and Panc-1 pancreatic adenocarcinoma cells were transfected with miR-302b-3p mimics or NC mimics. **a** The transfection efficiency of miR-302b-3p was analyzed by qRT-PCR. **b**, **c** The effects of miR-302b-3p on PC cell proliferation were determined by CCK8 and EdU assays. **d** Apoptosis of PC cells was analyzed by flow cytometry. **e** The migration and invasion abilities of PC cells were assessed by transwell assays. *P < 0.05, **P < 0.01

The effects of miR-302b-3p on the biological phenotypes of PC cells were further explored in PC cells transfected with miR-302b-3p mimics or negative controls. The transfection efficiency of miR-302b-3p was detected by qRT-PCR (Fig. 5a). The results of CCK8 and EdU assays indicated that overexpression of miR-302b-3p inhibited the proliferation of PC cells (Fig. 5b, c). Apoptosis assays revealed that miR-302b-3p overexpression induced PC cell apoptosis (Fig. 5d). miR-302b-3p overexpression attenuated PC cell migration and invasion (Fig. 5e).

### MiR-302b-3p targets SLC2A4 and inhibits its expression directly

**Fig. 6:**
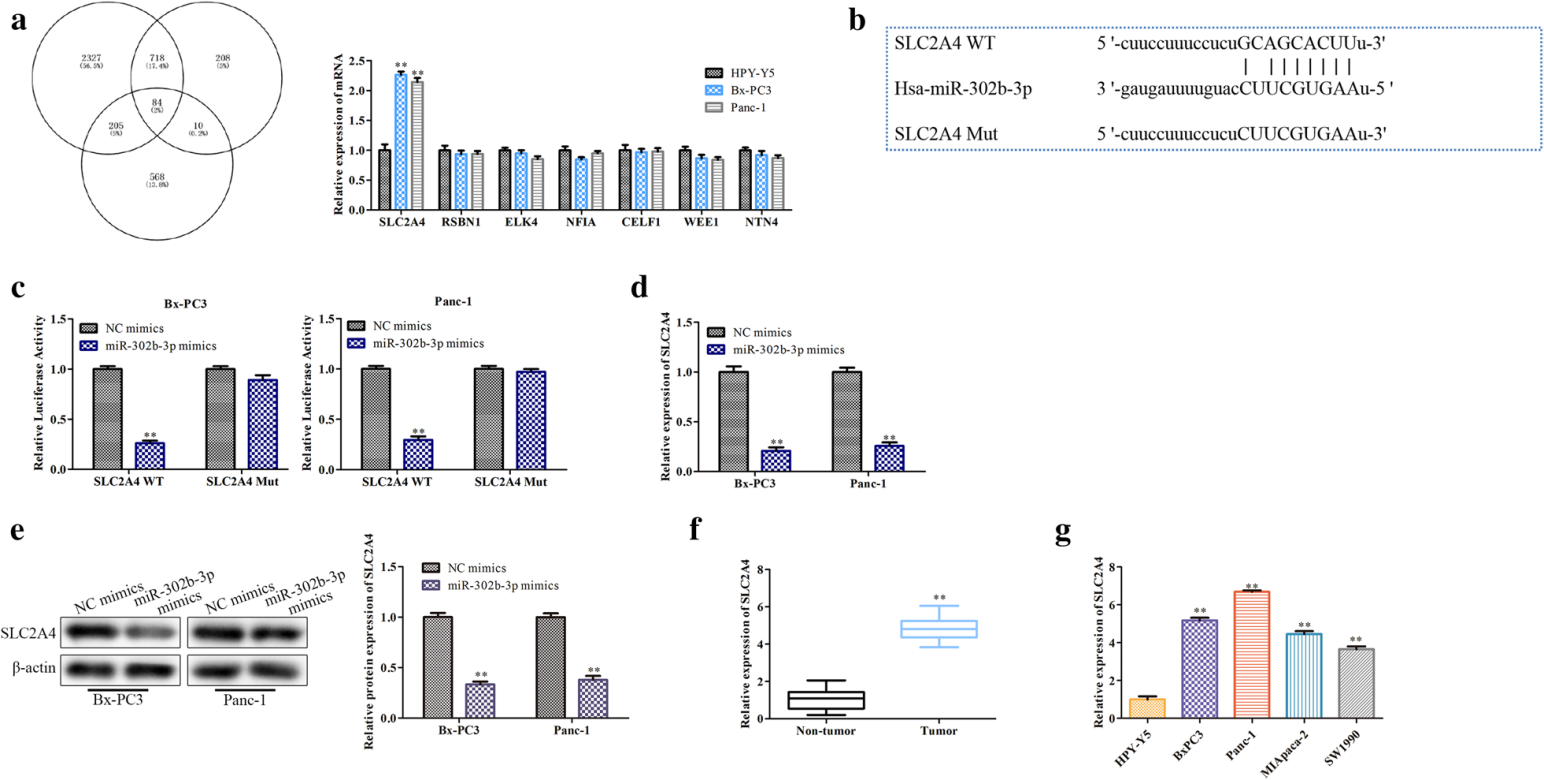
MiR-302b-3p targets SLC2A4 and inhibits its expression directly. **a** Identification of the potential targets of miR-302b-3p using online prediction databases and qRT-PCR analysis. **b** The predicted binding site of miR-302b-3p within the SLC2A4 3’UTR. **c** The binding interaction between SNHG16 and miR-302b-3p was confirmed by luciferase reporter assay. **d**, **e** qRT-PCR and western blot analyses of the effect of miR-302b-3p on SLC2A4 expression. **f**, **g** Relative expression levels of SLC2A4 in PC tissues and cell lines. *P < 0.05, **P < 0.01, ***P < 0.001

Three online tools (miRDB, TargetScanHuman 7.2 and starBase V2.0) were used to screen seven targets of miR-302b-: SLC2A4, RSBN1, ELK4, NFIA, CELF1, WEE1 and NTN4. Among these targets, SLC2A4 (solute carrier family 2 member 4) was the gene with the most significantly increased expression in PC cells (Fig. 6a). The putative site for miR-302b-3p and SLC2A4 binding is indicated in Fig. 6b. The dual luciferase reporter assay verified the targeted binding of miR-302b-3p to the 3’UTR of SLC2A4 mRNA (Fig. 6c). Moreover, transfection of miR-302b-3p mimics reduced the mRNA level, as well as the protein level, of SLC2A4 (Fig. 6d, e). In addition, elevated SLC2A4 expression was observed in PC tissues and cell lines (Fig. 6F and G). These data prove that miR-302b-3p targets SLC2A4 and inhibits its expression directly.

### SNHG16 promotes PC progression partially through the miR-302b-3p/SLC2A4 axis

**Fig. 7:**
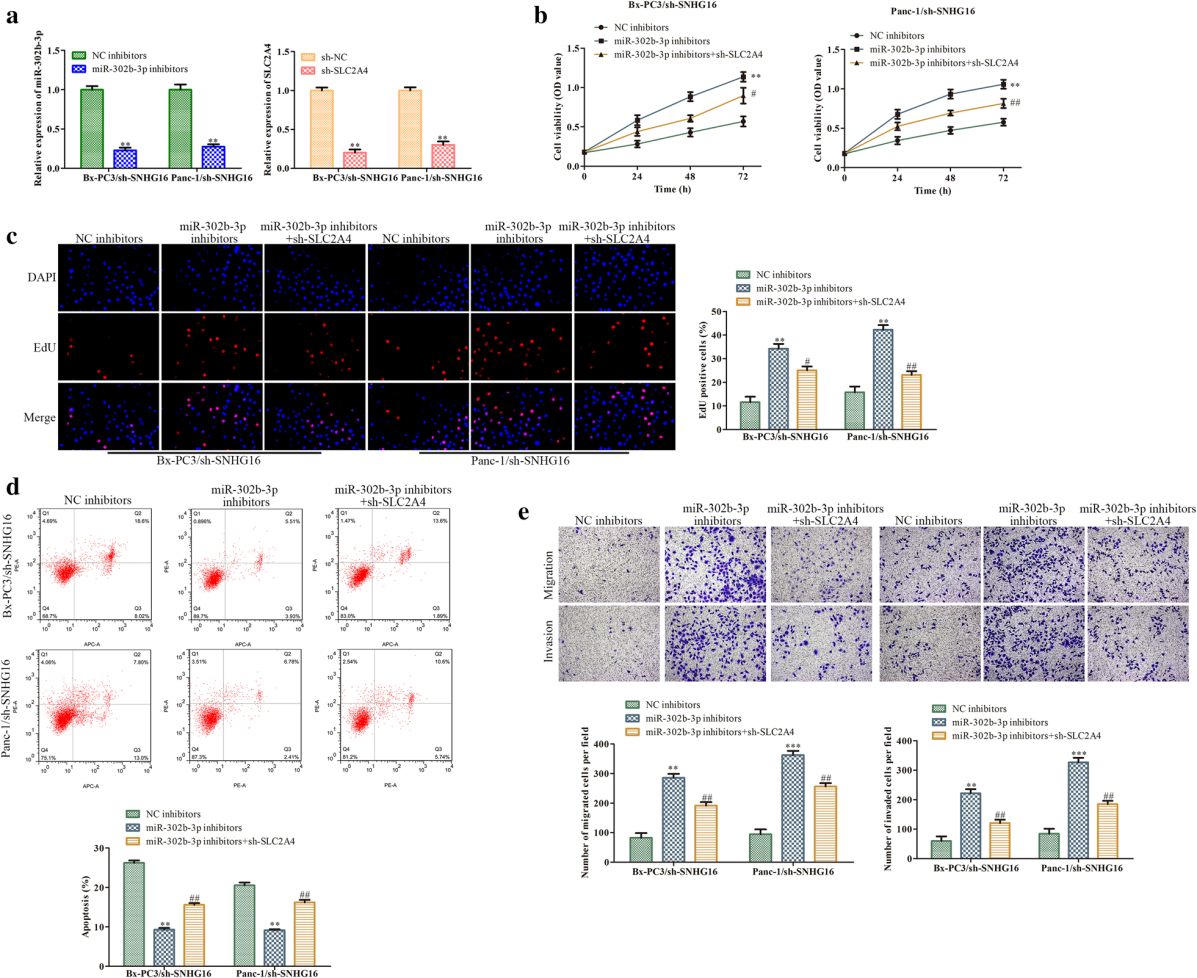
SNHG16 promotes PC progression partly via the miR-302b-3p/SLC2A4 axis. BxPC3 or Panc-1 cells with SNHG16 knockdown were transfected with the miR-302b-3p inhibitor, sh-SLC2A4, miR-302b-3p inhibitor plus sh-SLC2A4 or their corresponding negative controls. **a** The transfection efficiencies were evaluated by qRT-PCR. **b**, **c** Cell proliferation was assessed by CCK8 and EdU assays. **d** Apoptosis of BxPC3 or Panc-1 cells was detected by flow cytometry. **e** The migration and invasion abilities were evaluated by transwell assays. **P < 0.01 vs. NC inhibitor. ^#^*P* < 0.05, ^##^*P* < 0.01 vs. miR-302b-3p inhibitor

To confirm the correlation of the SNHG16/miR-302b-3p/SLC2A4 axis with PC progression, PC cells with stable silencing of SNHG16 were transfected with the miR-302b-3p inhibitor, miR-302b-3p inhibitor plus sh-SLC2A4, or NC inhibitor, and the biological phenotypes of these PC cells were analyzed. First, after transfection of Bx-PC3 and Panc-1 cells with sh-SNHG16, transfection of the miR-302b-3p inhibitor significantly inhibited miR-302b-3p expression, and transfection of sh-SLC2A4 decreased the expression of SLC2A4 (Fig. 7a). CCK-8 and EdU assays revealed that SLC2A4 knockdown reversed the promotion of PC cell proliferation induced by the miR-302b-3p inhibitor (Fig. 7b, c). The inhibition of apoptosis by the miR-302b-3p inhibitor was also abolished in PC cells cotransfected with the miR-302b-3p inhibitor and sh-SLC2A4 (Fig. 7d). Moreover, the increases in the migration and invasion abilities of PC cells with miR-302b-3p knockdown were suppressed by sh-SLC2A4 transfection (Fig. 7e). These results confirmed that SNHG16 participated in the progression of PC by targeting the miR-302b-3p/SLC2A4 axis.

## Discussion

PC is a highly aggressive solid tumor that frequently causes local invasion and early metastasis, which causes more than 300,000 deaths annually. The prognosis of PC patients is very poor, and the overall 5-year survival rate is less than 5% [15]. Therefore, it is important to identify promising diagnostic markers or targeted therapies to gradually inhibit the progression of PC. With the development of sequencing technology, an increasing number of noncoding RNAs have been discovered. Among noncoding RNAs, lncRNAs have received increasing attention due to their wide range of functions.

Small nucleolar RNA host gene 16 (SNHG16) has been reported as an oncogenic lncRNA in multiple cancers, such as colorectal cancer [16], non-small cell lung cancer [17], breast cancer [18], and clear cell renal cell carcinoma [19]. Further research indicated that SNHG16 constitutes a novel prognostic marker that promotes tumor formation and metastasis in vivo and in vitro by sponging miR-146a, further inducing MUC5AC expression in NSCLC [17]. Here, we investigated the novel biological effects of SNHG16 in PC.

The pivotal conclusion of this study is that SNHG16 plays a critical role in PC. The results demonstrated that SNHG16 is significantly upregulated in human PC tissues and PC cells, suggesting that this elevated lncRNA plays crucial roles in PC progression. Moreover, it was found that increased SNHG16 expression was associated with overall survival and indicated poor prognosis in patients with PC. This finding suggests that SNHG16 might be a prognostic marker for PC.

Functional experiments indicated the inhibitory effects of SNHG16 silencing on PC cell proliferation, migration and invasion and the promotive effect on apoptosis. Mechanistically, the target gene, miR-302b-3p, was predicted and identified by a luciferase reporter assay and RIP. MiR-302b-3p was highly expressed in both PC tissues and cells. Subsequent functional experiments confirmed that miR-302b-3p promoted the proliferation, migration and invasion of PC cells, effects opposite those of SNHG16. These data implied that SNHG16 acts as a sponge to negatively regulate miR-302b-3p expression in PC cells. Moreover, we investigated whether miR-302b-3p can directly target the 3’UTR of SLC2A4 and inhibit SLC2A4 expression in PC cells.

SLC2A4 is the gene encoding the insulin-sensitive glucose transporter GLUT4, which is an insulin-sensitive glucose transporter that plays a key role in glucose homeostasis [20]. SLC2A4 is an efficient glucose transporter that is located in cytoplasmic vesicles and can be transferred to the plasma membrane to take up glucose when stimulated by insulin [21]. It has been shown that SLC2A4 expression is elevated during cancer progression [22], 23], consistent with our results. SLC2A4 inhibition abolished the biological effects of miR-302b-3p downregulation on inducing the proliferation, migration and invasion of PC cells. Thus, we suggest that SNHG16 acts as an oncogene during PC progression by targeting the miR-302b-3p/SLC2A4 axis and that SNHG16 is expected to be a potential target for the early diagnosis and treatment of PC.

## Conclusions

1. Expression of the lncRNA SNHG16 is elevated in PC tissues and cell lines.
2. Knockdown of SNHG16 suppresses PC cell migration and invasion.
3. SNHG16 acts as a sponge to regulate miR-302b-3p expression in PC cells.
4. Overexpression of miR-302b-3p inhibits the proliferation, migration and invasion of PC cells and promotes their apoptosis.
5. MiR-302b-3p targets SLC2A4 and inhibits its expression directly.
6. SNHG16 promotes the progression of PC via the miR-302b-3p/SLC2A4 axis.
7. SNHG16 is expected to be a potential target for the early diagnosis and treatment of PC.

## Supplementary Information

**Additional file 1: Table S1.** Clinical pathologic features.

## Data Availability

All data generated or analyzed during this study are included in this published article.

## Abbreviations

PCPC: Pancreatic adenocarcinoma
lncRNAslncRNAs: Long noncoding RNAs
SNGH16: Small nucleolar RNA host gene 16S
DMEM: Dulbecco’s modified Eagle’s medium
