# Supplementary material for: LncRNA SNHG16 contributes to tumor progression via the miR-302b-3p/SLC2A4 axis in pancreatic adenocarcinoma
Source: Cancer Cell Int. 2021 Jan 12;21:51. doi: 10.1186/s12935-020-01715-9 (PMC7805184; doi:10.1186/s12935-020-01715-9)
Supplement: Supplementary file 1 — Additional file 1: Table S1. Clinical pathologic features. [file 12935_2020_1715_MOESM1_ESM.docx]

Table 1 Clinical pathologic features

|  | SNHG16 | |  |
| --- | --- | --- | --- |
| Clinical parameters | Low expression  (n=12) | High expression  (n=18) | P value |
| Age |  |  | 0.715 |
| >45 | 7 | 9 |  |
| ≤45 | 5 | 9 |  |
| Clinical stage |  |  |  |
| I~II | 5 | 11 | 0.692 |
| III~IV | 7 | 7 |  |
| Gender |  |  |  |
| Male | 8 | 10 | 0.059 |
| Female | 4 | 8 |  |
| Tumor size |  |  |  |
| >2 cm | 5 | 4 | 0.038* |
| ≤2 cm | 7 | 14 |  |
| Lymph node metastasis |  |  |  |
| Absent | 4 | 5 | 0.017* |
| Present | 8 | 13 |  |
